# Investigation of fullerene motion on thermally activated gold substrates with different shapes

**DOI:** 10.1038/s41598-022-18730-7

**Published:** 2022-08-24

**Authors:** Amir Shamloo, Mohammad Ali Bakhtiari, Mahdi Tohidloo, Saeed Seifi

**Affiliations:** School of Mechanical Engineering, Sharif University of Technology, Azadi Ave., Tehran, Iran

**Keywords:** Engineering, Materials science, Nanoscience and technology

## Abstract

In the current study, the regime of motion of fullerene molecules on substrates with different shapes at a range of specific temperatures has been investigated. To do so, the potential energy of fullerene molecules was analyzed using the classical molecular dynamics method. C_20_, C_36_, C_50_, C_60_, C_72_, C_76_, C_80_, and C_90_ fullerene molecules were selected due to their spherical shapes with different sizes. In addition, to completely analyze the behavior of these molecules, different gold substrates, including flat, concave, the top side of the step (upward step), and the downside of the step (downward step) substrates, were considered. Specifying the regime of the motion at different temperatures is one of the main goals of this study. For this purpose, we have studied the translational and rotational motions of fullerene molecules independently. In the first step of the investigation, Lennard-Jones potential energy of fullerene molecules was calculated. Subsequently, the regime of motion of different fullerenes has been classified, based on their displacement and sliding velocity. Our findings indicated that C_60_ is appropriate in less than $5\%$ of the conditions. However, C_20_, C_76_ and C_80_ molecules were found to be appropriate candidates in most cases in different conditions while they were incompetent only in seven situations. As far as a straight-line movement is considered, the concave geometry demonstrated a better performance compared to the other substrates. In addition, C_72_ indicated less favorable performance concerning the range of movement and diffusion coefficients. All in all, our investigation helps to understand the performance of different fullerene molecules on gold substrates and find their probable application, especially as a wheel in nano-machine structures.

## Introduction

Manipulation of nano-scale materials is becoming increasingly appealing for various technological objectives, thanks to the rapid development of nano-robots. In current years, quite a few transportation mechanisms have been suggested to carry nano-sized particles^1^. However, most of these approaches were incompetent due to several reasons. First, practically all created nano-manipulators are several orders of magnitude larger than their payload, which is contrary to natural nano- manipulators' performance^1,2^. In nature, molecules of the same order of magnitude or even smaller are capable of transporting atoms and molecules. Kinesin, for example, is a small protein that can transport quite large payloads properly^3,4^. Second, they are unable to simultaneously work on a large number of particles^2^.

James Tour et al. assembled several molecular motors with the goal of transporting other nano-scale materials^5–9^. These manufactured molecular machines have obtained such names like nanocars, nanotrucks, or other names because of their similarity to real cars among researchers^2,6,10^. Various nano-machines have been developed, each with a different shape and number of wheels. The first generation of synthesized nanocars moved with the aid of fullerene wheels^11,12^. C_60_ is a well-known molecule whose mobility on various substrates has been illustrated in a number of experimental and computational studies^12,13^. In addition, the motion of C_60_ on graphene, silicone, and gold substrates has been studied previously^14–17^. However, fullerene-wheeled nanocars have shown more profitable performance on the gold substrate due to their stability and conductivity^13^. Four or three-wheeled nano-machines with C_60_ as a wheel were fabricated significantly in previous studies of these nano-machines^5^. Vaezi et al.^18^ investigated the motion of C_60_ molecule on the boron-nitride substrate at different temperatures. They indicated that as the temperature increased, the rolling motion became more significant than the siding motion, and the range of the movement and diffusion coefficients became greater. Despite the progress made in the study of C_60_, the regime of motion of other fullerenes has not been investigated in detail. Thus, it seems extensively needed to investigate the mobility of other fullerene molecules on different substrates to assess their contingent applications. For instance, Wang et al.^19^ investigated the motion of C_60_, C_72_, C_180_, C_240_, and C_260_ fullerenes on the graphene substrate. They demonstrated that all molecules reached the end of the substrate with maximum velocity and started fluctuating at that point. Hence, these molecules may be utilized in the construction of high-frequency nano-switches, nanoparticle transport, or nano-robot components.

It is worthy to note that prior to utilizing these nano-machines, experimental or analytical approaches should be used to determine their class of motion^20^. Scanning Tunneling Microscopy (STM) is an effective measurement technique that has been utilized to monitor a range of nanocars^21^. Shirai et al.^9^ and Zhang et al.^20^ have employed experimental methods to evaluate the motion of nano-carriers. They studied the mobility of numerous fullerene-wheeled nanocars on a gold substrate at different temperatures. However, STM has a number of disadvantages, such as it requires an expensive and time-consuming process while just a few images can be obtained in a minute and the details of the motion can be partially displayed^22^. As a result, other approaches such as computational simulation can be suitable for measuring the motion of these nano-machines in various situations^23–27^. Akimov et al.^28^ and Konyukhov et al.^29,30^ simulated a nanocar with rigid C_60_ wheels and rigid chassis using the coarse-grained molecular dynamics method. Even though their simplified assumptions allowed them to perform simulations faster, it is essential to mention that the model precision was reduced in their study resulting in missing the details about the nanocar motion.

In this study, the motion of several fullerene molecules on four different gold substrates has been investigated. C_20_, C_36_, C_50_, C_60_, C_72_, C_76_, C_80_, and C_90_ fullerene molecules have been selected due to their spherical shapes with different sizes. In addition, to completely analyze the behavior of these molecules, different gold substrates, including flat, concave, the top side of the step (upward step), and the down side of the step (downward step) substrates, were considered. In the first step, the potential energy of fullerene molecules was calculated and then their variation during motion was investigated. Afterwards, the probable motion was predicted in different conditions based on the obtained results. The classical molecular dynamics method has been performed in the second step. The motion of fullerenes has been examined on the thermally induced substrates to achieve a better control over fullerene movement. Modelling the fullerene motion can result in anticipating the movements of fullerene-based nanocars under a myriad of methods.

## Methods

### Potential energy of fullerenes

**Figure 1:**
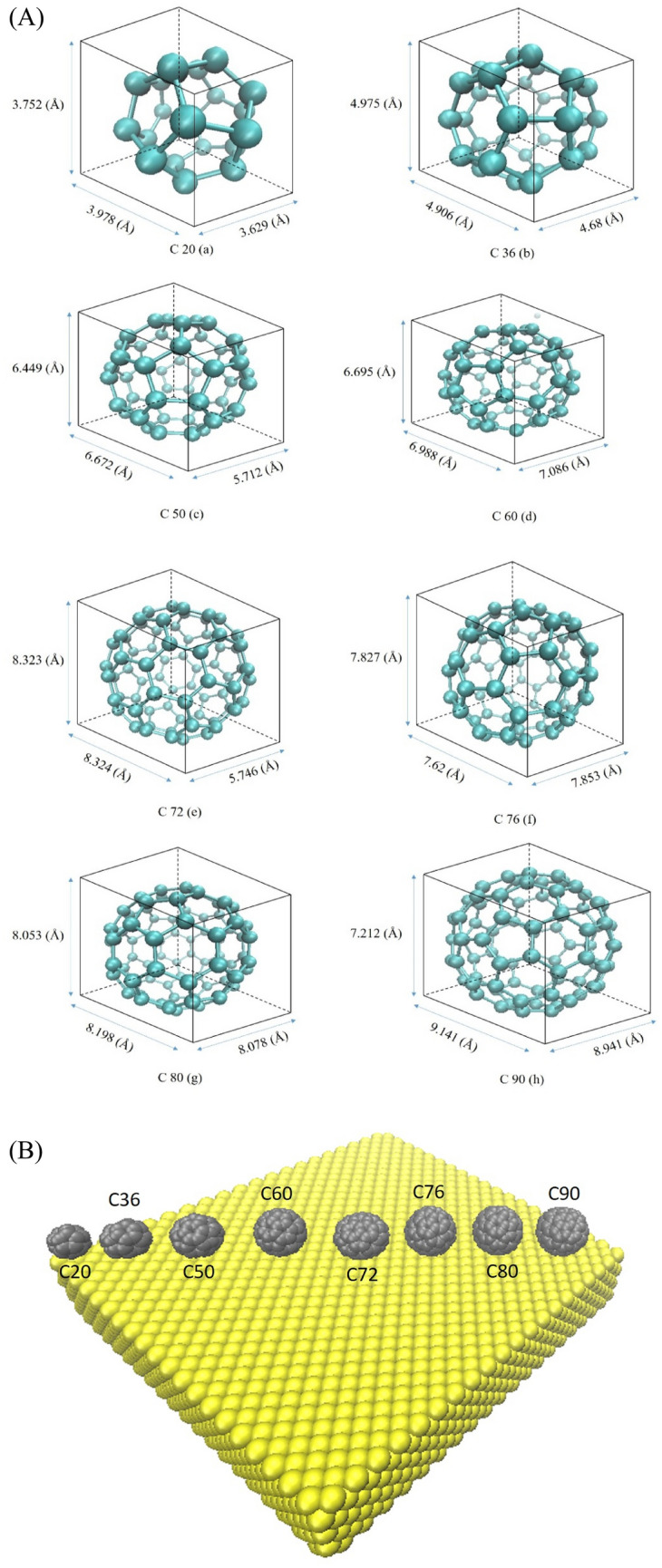
(**A**) The three-dimensional view of Different fullerenes simulated in this study which are C_20_ (a), C_36_ (b), C_50_ (c), C_60_ (d), C_72_(e), C_76_ (e), C_80_ (d) and C_90_ (h). (**B**) the fullerene molecules contacted from Hexa-Down orientation on the substrate at each starting point.

**Figure 2:**
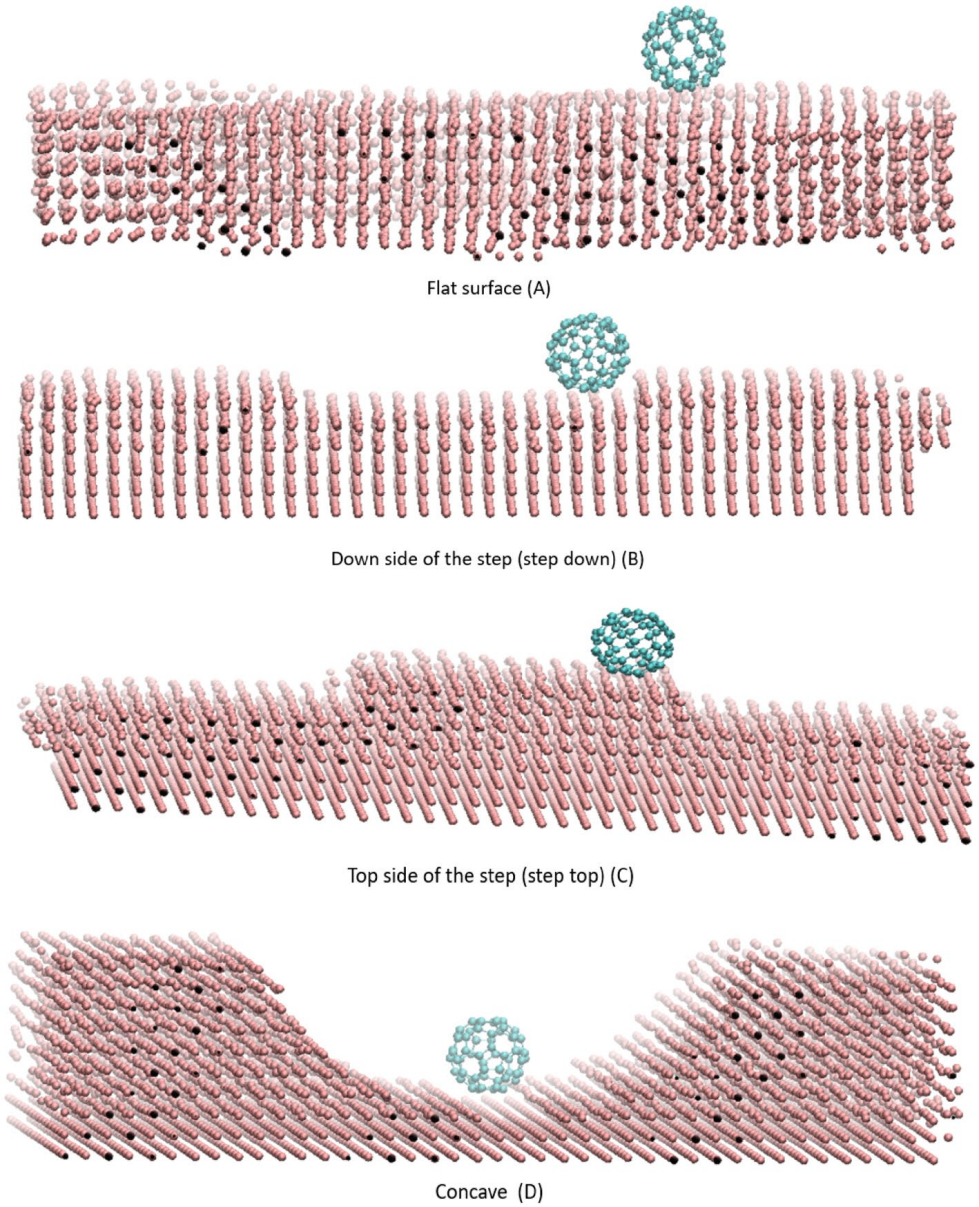
C_60_ fullerene on different gold substrates. (**A**, **B**, **C**, and**D**) representing the Flat surface, Down side of step, Top side of the step, and Concave substrates, respectively.

Potential energy analysis is a powerful method for predicting the mobility of fullerenes on quite a few substrates. This section investigates the mobility of several fullerenes (Fig. 1) on gold substrates with different shapes (Fig. 2), namely, the flat, upward step, downward step, and concave substrates. The upward and downward step substrates were considered to provide a better control over the motion of fullerene (Fig. 2 B,C). Furthermore, the central part of the concave substrate corresponds to the flat substrate, and its Up and down sides correspond to the downward step and upward step substrates when fullerenes are lowered or raised, respectively (Fig. 2D).

It is worth noting that in calculating the potential energy, fullerene is considered a rigid molecule. It means that the potential energy between the carbon atoms is constant and does not need to be calculated. Thus, the internal potential energy among the carbon atoms of fullerene was ignored, and the potential energy of fullerene stands for the potential energy resulting from the interaction between fullerene's carbon atoms and the gold atoms of the substrate. The rigid molecular structures of all fullerenes were provided from the Nanotube Modeler program in the fullerene library^31^. Some fullerene molecules have many isomers, so we select the one that has a spherical shape like C_60_ (e.g. C_36_ has $15$ isomers and we chose the isomer named No.15-D6h.cc1**,** C_50_ has $271$ isomers and we chose the isomer named No.271-D5h.cc1**,** C_76_ has $2$ isomers and we chose the isomer named C76-Td.cc1, C_80_ has $7$ isomers and we chose the isomer named No.6-D5h.cc1, and C_90_ has $46$ isomers and we chose the isomer named No.20-C1.cc1 based on their spherical shapes, from the nanotube modeler).

According to Pishkenari et al.^32^, the potential energy of C_60_ on a gold substrate varies depending on its orientation. They compared four different C_60_ orientations, taking into account translational and rotational motion, and found that the Hexa-down orientation has the most stable motion. Therefore, in the current work for all the aforementioned fullerenes were placed on substrates via their Hexa-down orientation except C20, which was placed on its pentagonal side.

The potential energy of fullerenes was calculated employing Lennard-Jones potential as below (Eq. 1):

1 $$E_{Lj} = 4\varepsilon \left[ {\left( {\frac{\sigma }{r}} \right)^{12} - \left( {\frac{\sigma }{r}} \right)^{6} } \right]$$

where $\sigma$, $\varepsilon$, and $r$ are potential parameters that indicate the equilibrium distance of the ${\text{Au}}{-}{\text{C}}$ bond $(\sigma = 2.9943 {\text{\AA}})$, the well depth of the potential ($\varepsilon = 0.01273 eV$), and the distance between the gold and carbon atoms in an equilibrium location, respectively. In addition,$r_{cut - off}$ represents the cut-off radius for the Lennard-Jones potential and is set to $13 {\text{\AA}}$.

### Simulation setup

In the current study, the motion of several fullerene molecules on the gold surfaces with different shapes has been examined using the classic molecular dynamics method. Simulations were performed at different temperatures in the range of $75K$ to $600K$ to investigate the effect of the temperature on the mobility of fullerene molecules. The temperature of the substrate and fullerenes was regulated by employing the Nose–Hoover thermostat. The fullerene molecule was placed on the top of the substrate, while the lower layer was supposed to be rigid. Substrate size was adjusted to $18a\times 18a\times 3a$, where a stands for the gold lattice constant, set to be $4.078{\text{ {\AA}}}$. Additionally, periodic boundary conditions were utilized in the $x$ and $y$ directions, and the plane direction of the gold surface was considered to $(001)$ concerning the FCC crystalline direction.

To model the interaction between the gold atoms in the substrate, the EAM potential was applied^33^. This potential was developed using density functional theory, which is recognized as one of the most accurate potentials for mimicking Au and other FCC metals so far. This potential has been shown to accurately replicate the vacancies and disorders in $\mathrm{Au}$ substrates, and the dislocation structures predicted by this potential match with experimental observations properly. Simulations were performed using LAMMPS software^34^, and the results are envisioned by operating the VMD package^35^. Before starting the simulation, the system was relaxed for 200,000 steps, and then, the simulation was performed for $8 ns$ considering $1 fs$ time steps to attain accurate results. The velocity Verlet algorithm was used to integrate the equations of motion with a $1 fs$ time step. It is worth mentioning that $Tdamp$ is set to $50 fs$ in the LAMMPS software for NVT ensemble as well^36^.

## Results

**Figure 3:**
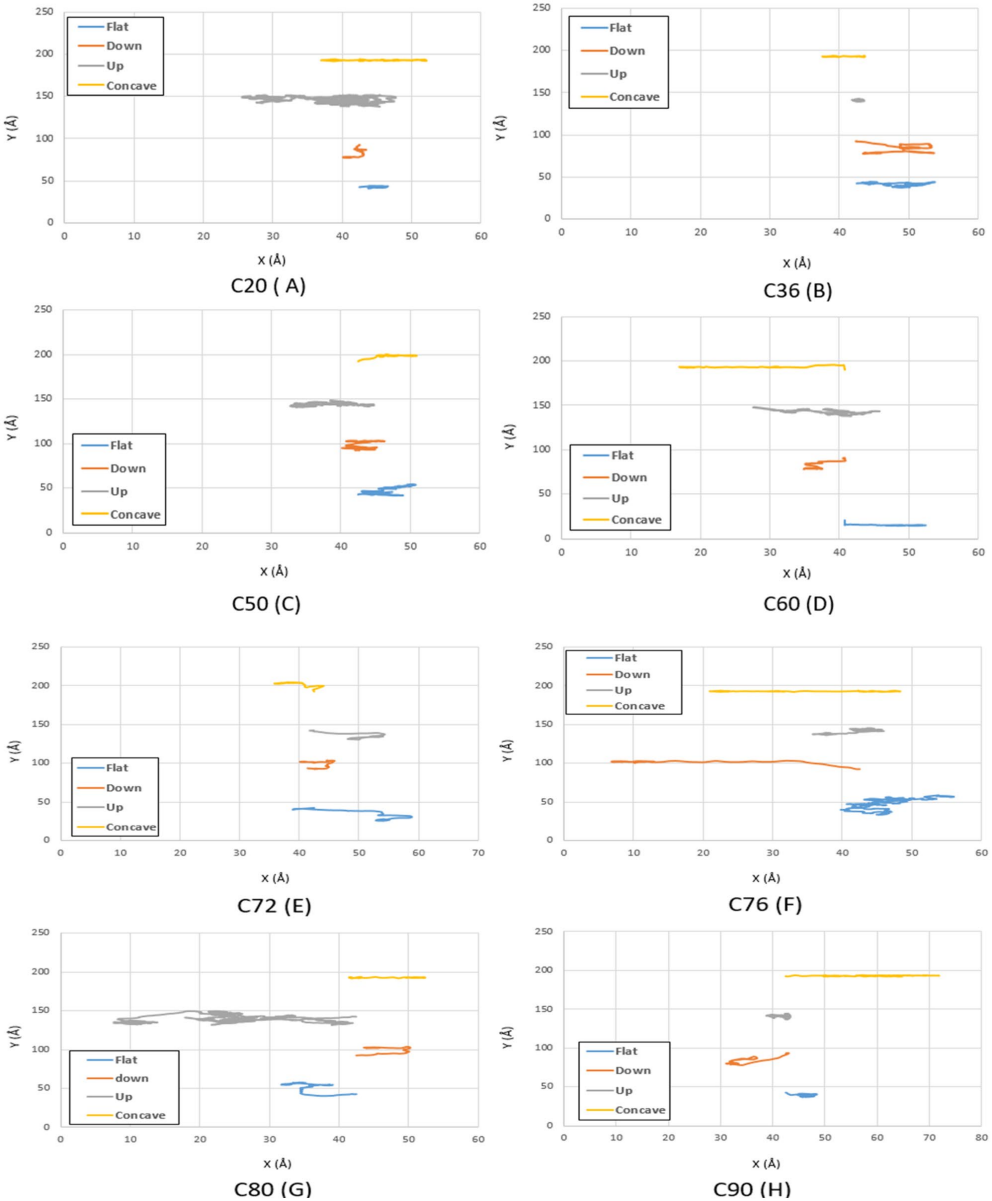
Trajectories of different fullerenes on a gold substrate at $75K$ for C_20_ (**A**), C_36_ (**B**), C_50_ (**C**), C_60_ (**D**), C_72_ (**E**), C_76_ (**F**), C_80_ (**G**), and C_90_ (**H** . The blue, red, black and yellow lines show the trajectories of the molecules on the flat, down side of the step, top side of the step, and concave substrates, respectively.

**Figure 4:**
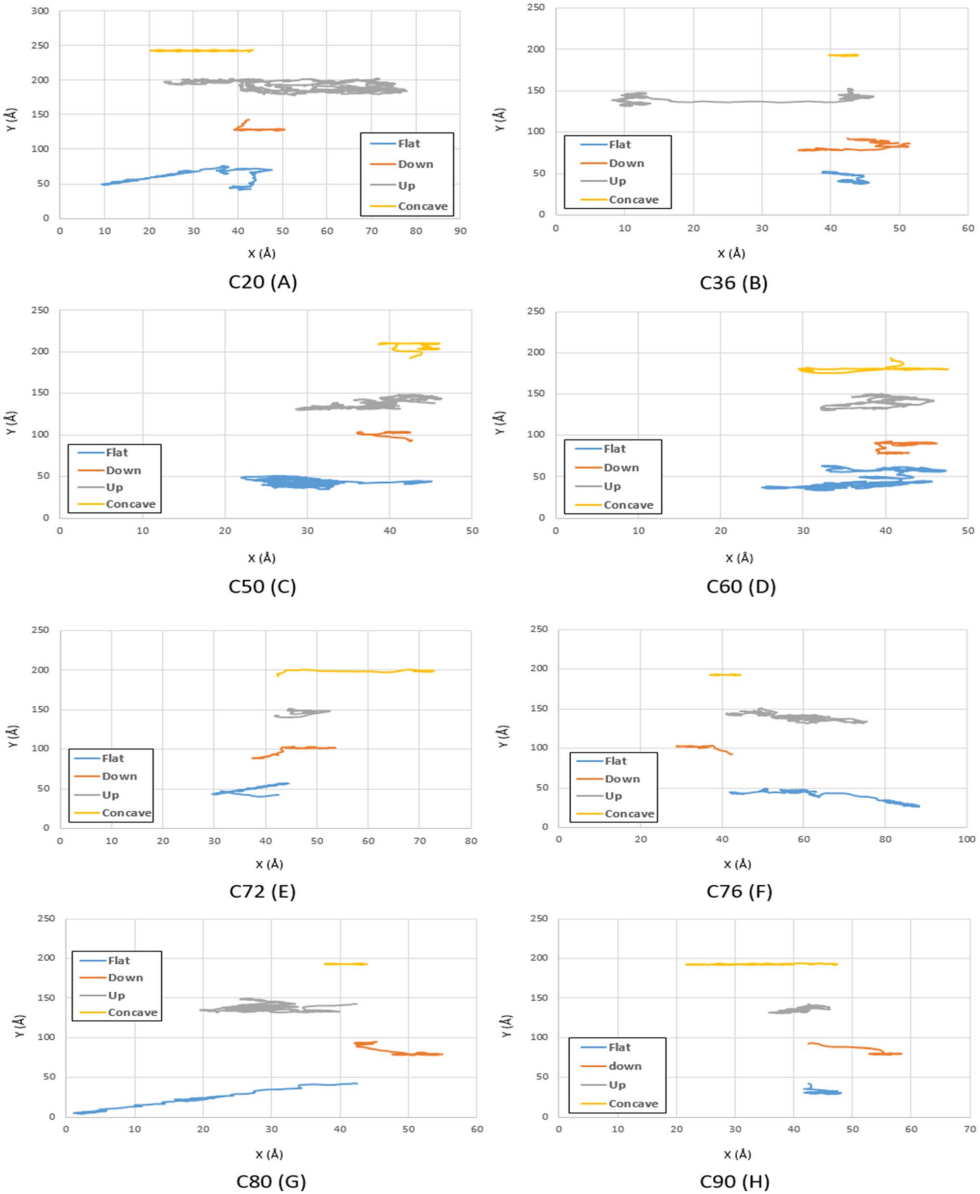
Trajectories of different fullerenes on a gold substrate at $150\mathrm{K}$ for C_20_ (**A**), C_36_ (**B**), C_50_ (**C**), C_60_ (**D**), C_72_ (**E**), C_76_ (**F**), C_80_ (**G**), and C_90_ (**H**). The blue, red, black and yellow lines show the trajectories of the molecules on the flat, down side of the step, top side of the step, and concave substrates, respectively.

**Figure 5:**
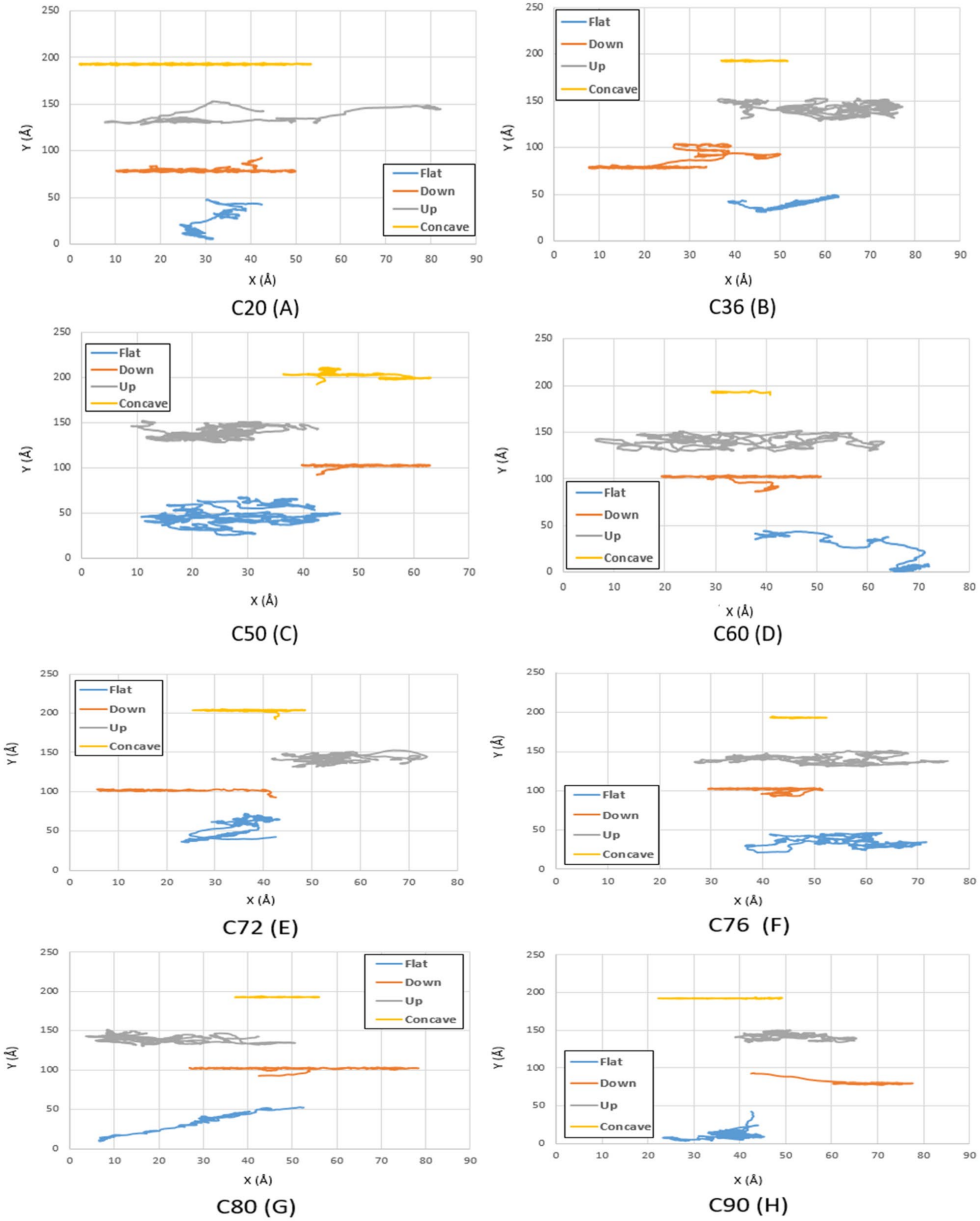
Trajectories of different fullerenes on a gold substrate at $300\mathrm{ K}$ for C_20_ (**A**), C_36_ (**B**), C_50_ (**C**), C_60_ (**D**), C_72_ (**E**), C_76_ (**F**), C_80_ (**G**), and C_90_ (**H**). The blue, red, black and yellow lines show the trajectories of the molecules on the flat, down side of the step, top side of the step, and concave substrates, respectively.

The main goal of this study is to find the optimal fullerene molecules to be used as a wheel in nano-machines. For this aim, the motion of fullerene molecules on different gold substrates has been analyzed in the first stage. According to the geometries studied in the current investigation, the fullerene molecules have shown more deviation from their direct path on the flat substrate than the other substrates since the motion of the molecules was not restricted on this substrate. After the flat substrate, the most deviation of the fullerene molecules happens on the upward step substrate due to its high-energy points resulting from the surface effects, and no energy is needed to jump down the step. Furthermore, by increasing the radius of the fullerene molecule from C_20_ to C_90_, it was observed that the range of motion of fullerenes has become smaller due to the generation of more non-bonded forces between the gold substrate and the fullerenes' carbon atoms (Figs. 3, 4 and 5 blue and gray lines).

**Figure 6:**
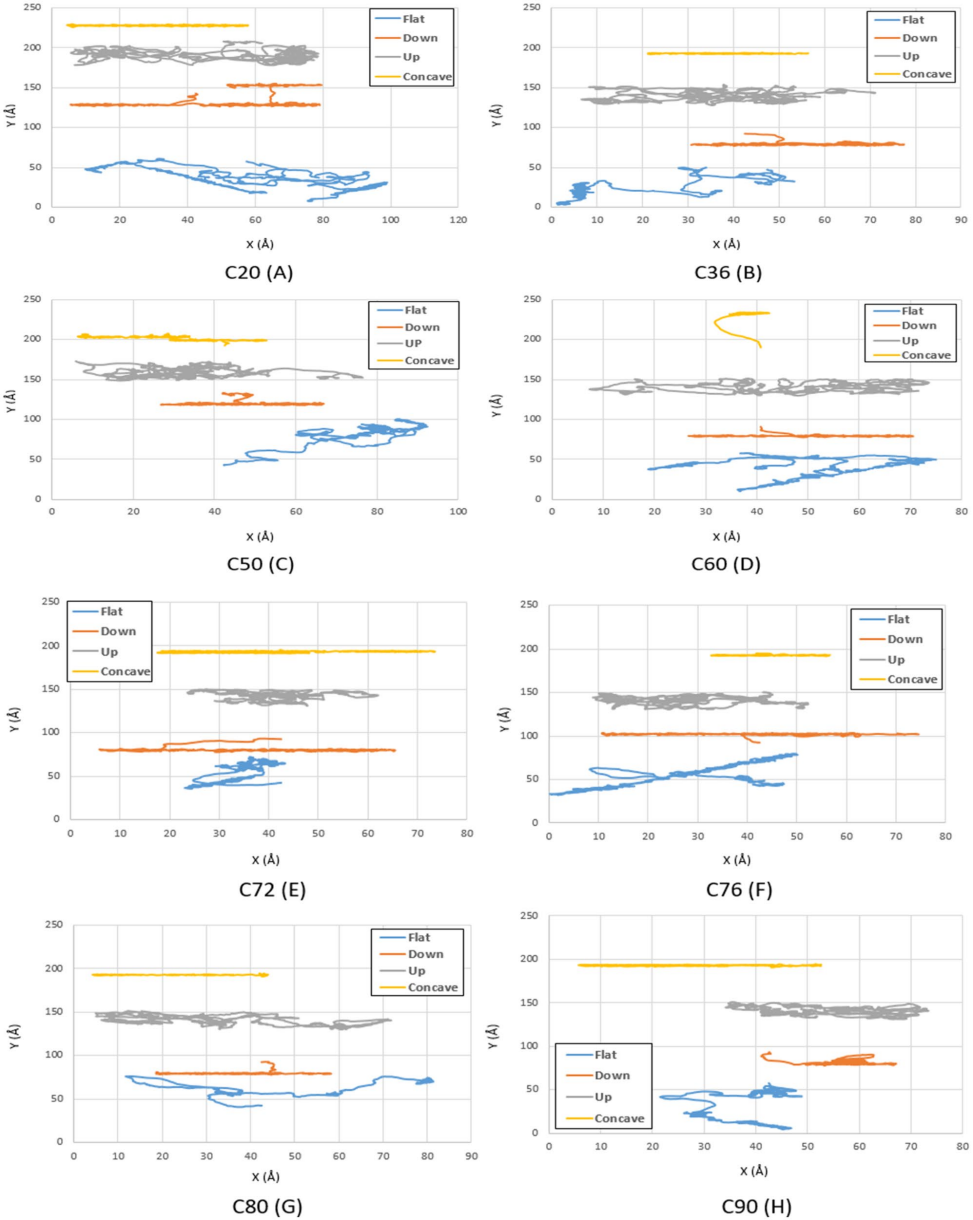
Trajectories of different fullerenes on a gold substrate at $400\mathrm{K}$ for C_20_ (**A**), C_36_ (**B**), C_50_ (**C**), C_60_ (**D**), C_72_ (**E**), C_76_ (**F**), C_80_ (**G**), and C_90_ (**H**). The blue, red, black and yellow lines show the trajectories of the molecules on the flat, down side of the step, top side of the step, and concave substrates, respectively.

**Figure 7:**
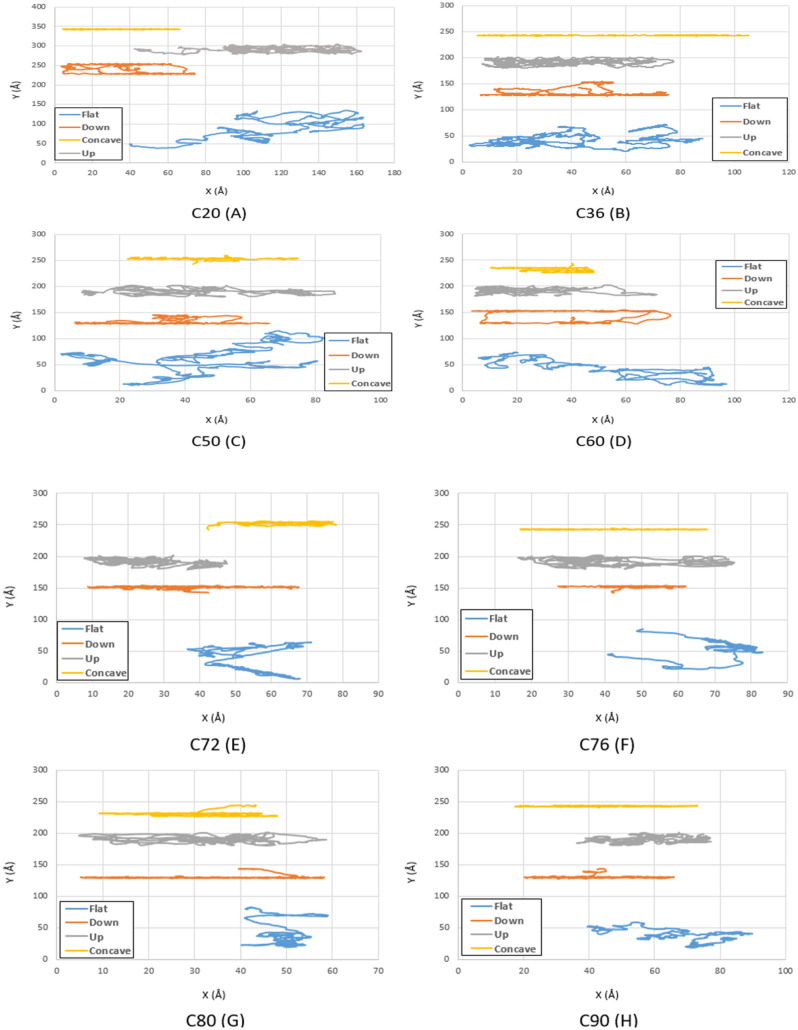
Trajectories of different fullerenes on a gold substrate at $500\mathrm{K}$ for C_20_ (**A**), C_36_ (**B**), C_50_ (**C**), C_60_ (**D**), C_72_ (**E**), C_76_ (**F**), C_80_ (**G**), and C_90_ (**H**). The blue, red, black and yellow lines show the trajectories of the molecules on the flat, down side of the step, top side of the step, and concave substrates, respectively.

**Figure 8:**
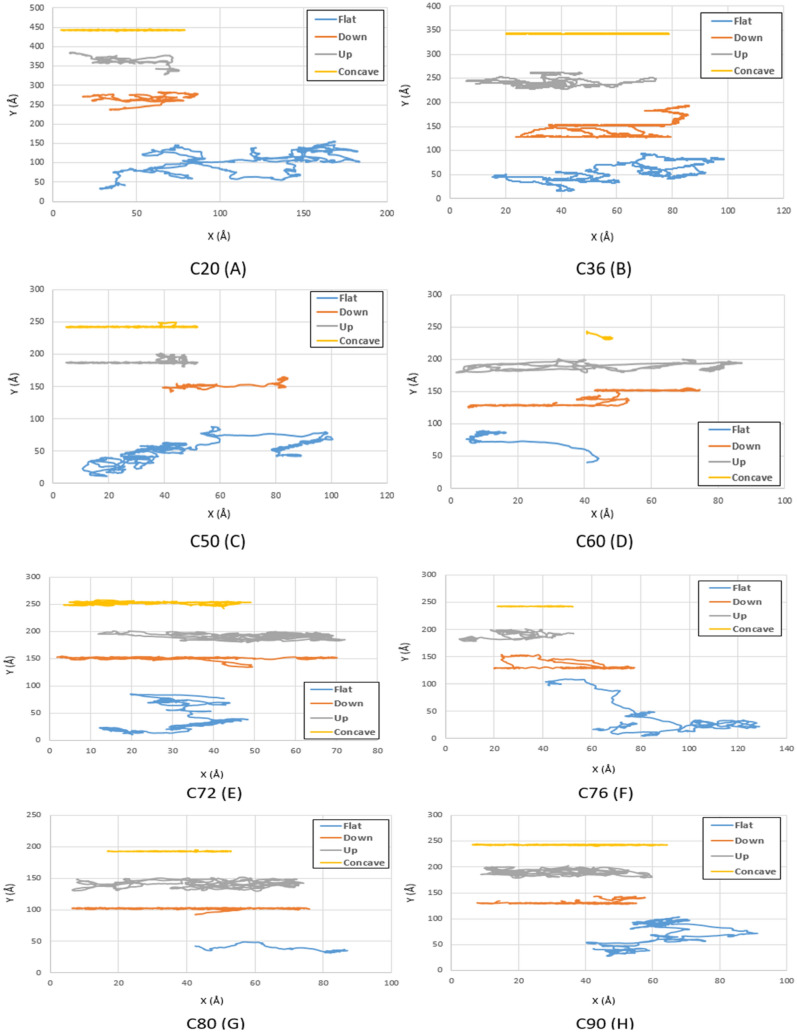
Trajectories of different fullerenes on a gold substrate at $600\mathrm{K}$ for C_20_ (**A**), C_36_ (**B**), C_50_ (**C**), C_60_ (**D**), C_72_ (**E**), C_76_ (**F**), C_80_ (**G**), and C_90_ (**H**). The blue, red, black, and yellow lines show the trajectories of the molecules on the flat, down side of the step, top side of the step, and concave substrates, respectively.

Contrary to the radius effects, increasing the temperature leads to a long-range movement for fullerenes due to increases in the internal energy of the molecules, which could overcome the non-bonded forces properly (Figs. 6, 7 and 8 blue and gray lines). Likewise, the downward step substrate has high-energy points, especially in the edges, which cause deviation of the molecules in this geometry. However, minimum energy is required for molecules to jump over the step; hence, fullerenes have shown less deviation on the downward step geometry than the upward step geometry. At low temperatures, long-range movements were not observed so much so that all fullerenes were merely fluctuating (Figs. 3 and 4 red lines). As the fullerenes enlarge, the energy required to jump up the steps increases; thus, larger molecules could overcome the fluctuating motion and commence their motion at high temperatures with few deviations (Figs. 7 and 8 red lines). Besides, by increasing the radius of fullerene from C_20_ to C_90_, the range of motion has increased on the downward step substrate. The same behavior has been repeated in the concave geometry. The higher the temperature, the greater range of fullerene motion was observed, especially when the radius and temperature increase simultaneously. It is worth mentioning that all fullerenes traveled a direct path at almost all temperatures due to several reasons. To begin with, the energy of fullerene molecules was devoted to climbing the gradual step form of the concave substrate. Second, due to the similar shape of the concave substrates and fullerenes, the movement of fullerenes is conducted in one direction. Third, Due to the fact that the concave substrate not only takes benefit of the long-range movement of fullerene on the flat substrate but also can successfully reduce the fullerene deviation because of the existence of a step-like surface on its both sides.

Moreover, a long-range movement has been observed for the concave substrate because of earlier mentioned reasons and the reciprocal effects of the temperature and radius on the motion of molecules, whose investigation is beyond the scope of this study (Figs. 3, 4, 5, and 6 yellow lines).

**Figure 9:**
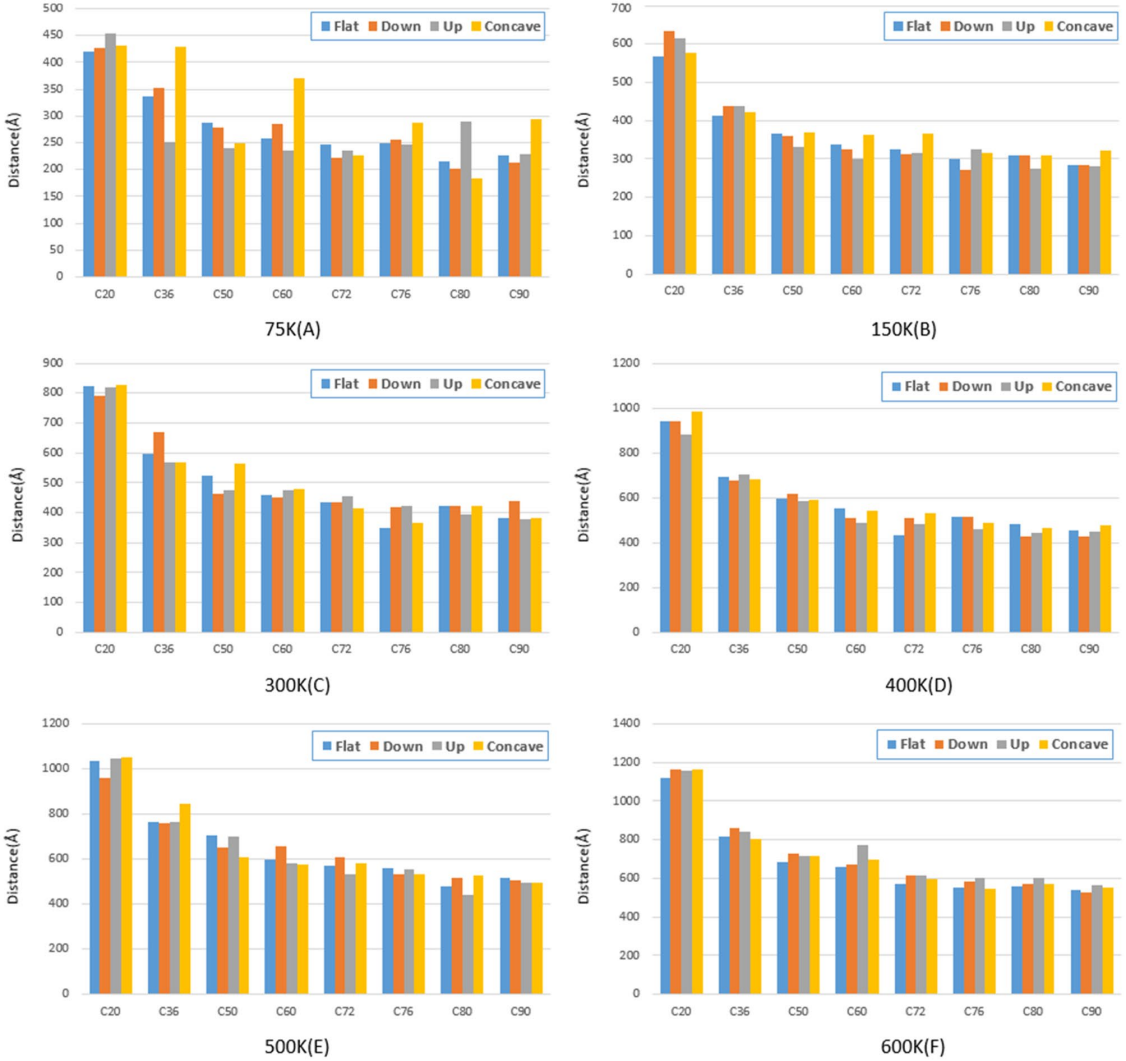
Traveled distance by fullerenes on a gold substrate for $75 \mathrm{K}$(**A**), $150 \mathrm{K}$ (**B**), $300 \mathrm{ K}$ (**C**), $400 \mathrm{K}$ (**D**), $500 \mathrm{K}$ (**E**), and $600 \mathrm{K}$ (**F**). The blue, red, black and yellow bars show the traveled distant of the molecules on the flat, down side of the step, top side of the step, and concave substrates, respectively.

**Figure 10:**
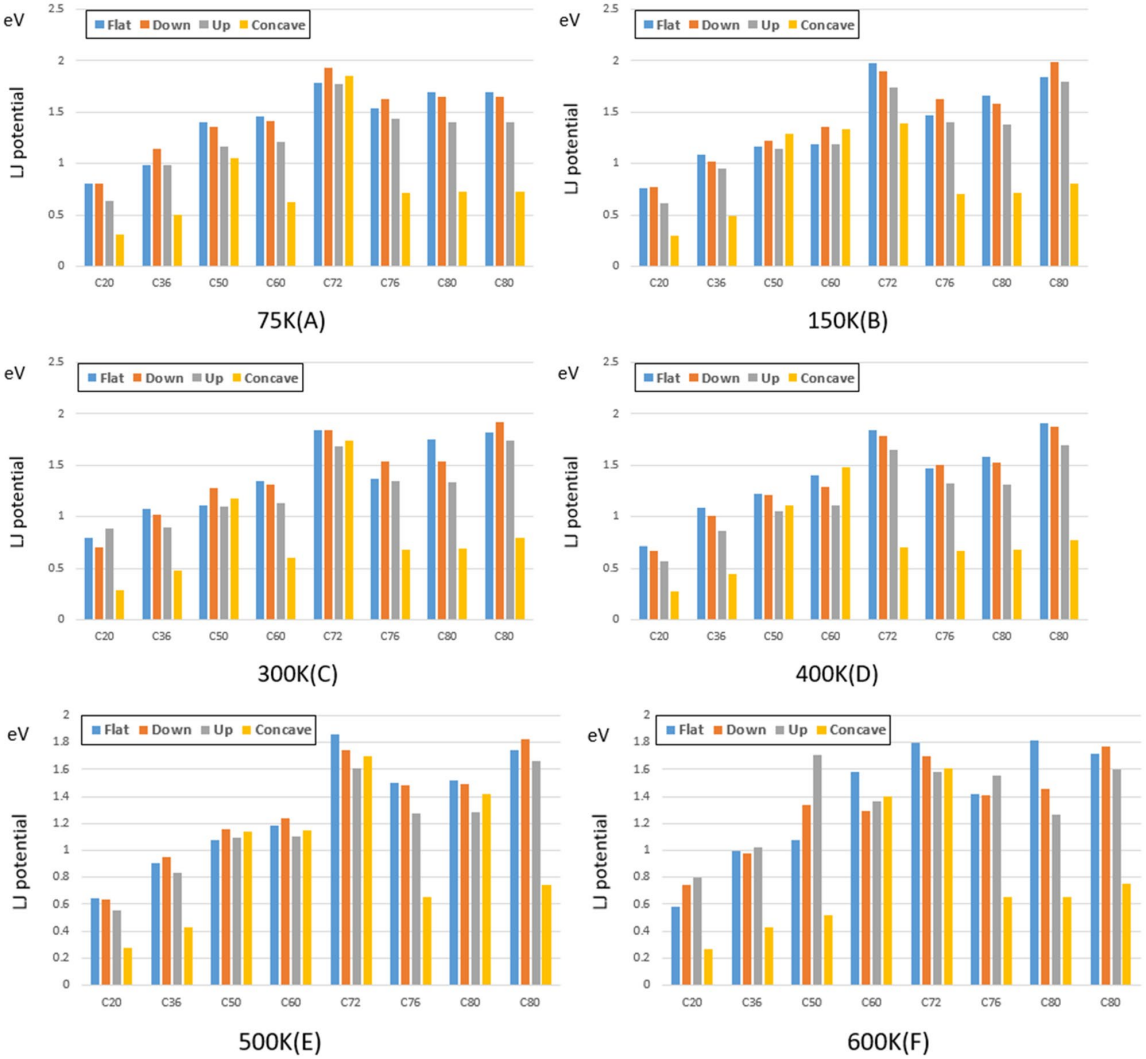
Lenard Jones potential energy for different fullerene molecules on a gold substrate for $75 K$ (**A**), $150 K$ (**B**), $300 K$ (**C**), $400 K$ (**D**), $500 K$ (**E**), and $600 K$ (**F**). The blue, red, black and yellow bars show Lenard Jones potential energy of the molecules on the flat, down side of the step, top side of the step, and concave substrates, respectively.

**Figure 11:**
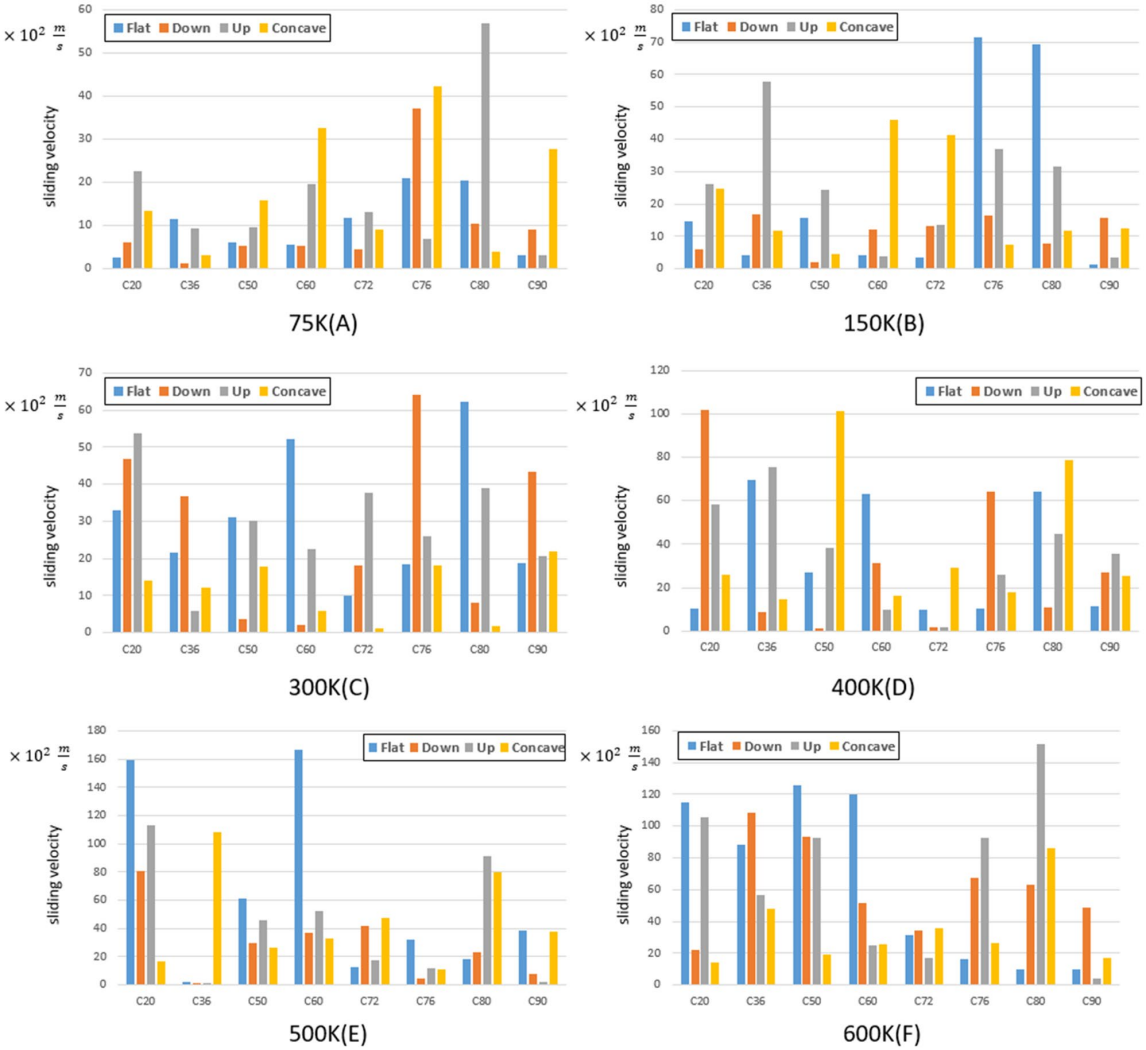
Sliding velocity for different fullerene molecules on a gold substrate for $75 K$ (**A**), $150 K$ (**B**), $300 K$ (**C**), $400 K$ (**D**), $500 K$ (**E**) and $600 K$ (**F**). The blue, red, black and yellow bars show sliding velocity of the molecules on the flat, down side of the step, top side of the step, and concave substrates, respectively.

To investigate the behavior of fullerene molecules on different gold substrates, the energy of Lennard-Jones, the distance traveled, and the sliding velocity of each fullerene are presented in Figs. 9, 10, and 11, respectively. The sliding velocity has been considered since the main factor of fullerenes’ movement is supplied by sliding motion rather than rolling motion.

The distance traveled by the molecules was increased as the temperature increased in all conditions (Fig. 9). In addition, a declining trend for the distance traveled by the molecules has been observed when the fullerenes' radius increased since as the fullerene enlarged, more carbon atoms interacted with the gold surface, slowing down the motion (Fig. 9). However, this decreasing trend for all temperatures has led to a constant value after C60 molecule. This stems from the fact that more carbon atoms were associated with the gold surface as the fullerene size increased, but their interactions will change slightly (Fig. 9).

As shown in Fig. 10, the higher the energy between the fullerenes and the gold surface, the less mobility was onbserved for fullerene molecules. Thus, the same Lennard-Jones potential energy for fullerenes on the flat, upward step, and downward step substrates has been observed. Concerning the concave substrate, less energy was observed due to the shape similarity of the gold surface and fullerenes (Fig. 10). However, C_72_ was the only exception, demonstrating the same potential energy on all the substrates due to its non-spherical shape.

The sliding velocity is one of the most important parameters for selecting fullerenes on different substrates, presented in Fig. 11 for all temperatures. As all the nanocars have been assembled with C_60_ wheels so far, only fullerenes indicated a better performance compared to C_60_ were investigated in the current study.

Given Fig. 3a, some of the fullerene molecules revealed more movements compared to C_60_ at $75K$, two of which are related to C_76_ on the downward step and concave substrates, and one of them is associated with C_80_ on the upward step substrate. Considering the aforementioned deviation results, The C_76_ molecule can be identified as the most desirable fullerene at $75K$.

At $150K$, despite the high mobility of C_76_ and C_80_ fullerenes on the flat substrate, they cannot be considered as alternative options due to the high amount of deviation on the flat substrate. Thus, C_60_ fullerene is considered as the best alternative for C_60_ at $150 K$ due to its higher momentum compared to other fullerenes. However, for applications in which fullerenes move on concave-like geometries, C_60_ fullerene is still recommended (Fig. 11B).

Regarding Fig. 11C, C60 molecule did not perform properly at $300 K$, and in accordance with the surface shape and mobility considerations, C_76_ molecule was introduced as the most suitable candidate at this temperature. It should be considered that the word "Candidate" was used for fullerenes that may have a better performance compared to C60 in different conditions.

At $400 K$, C_60_ has shown insufficient performance on all the substrates except on the flat substrate in which considerable deviations limited all the applications. Hence, two of the fullerene molecules suggested, including C_20_ and C_50_, revealed a good performance on the downward step and concave substrate, respectively. It should noted that C_80_ fullerene could also be considered a substitute for C_50_ molecule on concave geometry (Fig. 11D).

Although the performance of C_60_ molecule was unfavorable at $500 K$, other molecules also have not offered a more satisfactory performance in all conditions. However, C_20_ and C_60_ molecules are recommended due to their better mobility to be utilized on the flat substrates despite the high deviations. In addition, C_20_ on the upward and downward step substrates, and C_36_ on the concave substrates are vigorously suggested, because of their stable motions with fewer deviations (Fig. 11E).

At $600 \mathrm{ K}$, C_80_ molecule indicated a proper performance on the upward step substrate and has been recognized as the leading candidate instead of C_60_. After C_80_, other fullerenes also showed an acceptable performance, including C_20_ on the flat and upward step substrates, C_36_ on the flat and downward step substrates, C_50_ on the flat and upward/downward step substrates, and C_76_ on the upward/downward step substrates (Fig. 11F).

As a result, Table 1 represents the most desirable fullerene on all substrates and at all temperatures. According to this, the required nano-machines can be designed appropriately to gain the best performance in different conditions. Moreover, C_60_ molecule, which was considered as a wheel in all nano-machines, could be a candidate among the fullerenes only in a few cases. Therefore, the presented study can be considered a compelling research in connection with introducing the most desirable fullerene molecules under different conditions.

**Table 1:** Candidate fullerenes in different conditions.

	Flat	Up	Down	Concave
75 K	C76,C80	C80	C76	C76
150 K	C76,C80	C36	C36,C76,C90	C60
300 K	C60,C80	C20	C76	C50,C76,C90
400 K	C36,C60	C36	C20	C50
500 K	C20,C60	C20	C20	C36
600 K	C20,C50,C60	C80	C36,C50	C80

## Conclusion

Simulation can be a valuable tool for investigating physical/chemical phenomena^37–42^. The current study investigates the different fullerenes' motion on a flat, upward step, downward step, and concave substrates at $75 K$, $150 K$, $300 K$, $400 K$, $500 K$, and $600 K$. For this aim, the C_20_, C_36_, C_50_, C_60_, C_72_, C_76_, C_80_, and C_90_ fullerene molecules were chosen due to their shape and radius differences. Due to the fact that the nano-machine wheels play an important role in the nano-machine motion because they have the most interactions with the underneath substrate while they are connecting the nano-machine chassis. Hence, the current investigation can suggest optimal wheels for nano-machine movements and potentially improve the nano-machine performance for different applications.

As far as a straight-line movement is concerned, the fullerenes demonstrated the most deviation on the flat substrates compared to other substrates. At the same time, the concave, upward step, and downward step indicated fewer deviations due to their capability to restrict the unfavorable motions, respectively. Furthermore, as the temperature increased, the deviation of the fullerenes increased significantly except in the case of the concave substrate due to the restriction of fullerenes unlike the motion from both sides.

In the next stage, the effect of the radius on the fullerenes' motion was studied so that a long-range movement was observed for fullerenes on the concave substrate even at low temperatures ($75 K$ and $150 K$). However, the temperature has played a predominant role in the motion of the fullerene molecules on the flat, upward step, and downward step substrates.

The Lennard-Jones potential has been employed to evaluate the Van Der Waals interaction forces between the fullerenes and the gold surfaces. The result indicates that fullerenes showed the same Lennard-Jones potential energy on the flat, upward step, and downward step substrates at a constant temperature since the gold surfaces interacting with the carbon atoms had an identical structure. Nonetheless, for the concave substrate, in the light of its shape resemblance with fullerenes, carbon atoms have more interactions with the gold surface, which leads to greater Lennard-Jones potential energy. Moreover, the Lennard-Jones potential differences between the concave geometry and the other substrates become more significant when the fullerenes radius increases (from C_20_ to C_90_).

As previous studies stated, the temperature is the only effective characteristic of the distance traveled by the fullerenes, such that the surface changes did not influence the amount of the distance traveled. However, our findings revealed that as the radius increased, the distance traveled was decreased for the fullerenes at the same temperature. This stems from the fact that more carbon atoms were involved in the interaction with the gold surface, which slows the movement of fullerenes.

The sliding velocity parameter was also evaluated to determine the appropriate fullerenes according to probable nano-machines conditions (the dimension and temperature). To do so, more details are provided in Table 1, in which the candidate fullerenes are introduced considering the temperature and substrate changes. C_60_ molecule, which has been used as a wheel for nano-machines in almost all studies, was not recognized as a suitable candidate on the upward and downward step substrates at all temperatures. Besides, a better candidate was introduced instead of C_60_ on the flat substrate at all temperatures. The concave substrate at $150 K$ was the only case where no qualified candidate was discovered among our studied molecules. Our findings indicates that C_60_ is appropriate in less than $5\%$ of fullerenes objectives. C_20_, C_76_, and C_80_ molecules have been candidates in most cases in different conditions, such that they were incompetent only in seven situations, which is mentioned in the result section. In addition, the C_72_ molecule was the only fullerene that was not introduced as a candidate due to its cylindrical structure, which caused the molecule to fall on its more extensive cross-section after a period. All in all, our investigation helps to understand the optimal performance of different fullerene molecules on gold substrates in order to find their probable application, especially in nano-machines objectives.

## Data Availability

The data of this study is available upon reasonable request from the corresponding author.
